# Progress of emerging non-volatile memory technologies in industry

**DOI:** 10.1557/s43579-024-00660-2

**Published:** 2024-11-07

**Authors:** Markus Hellenbrand, Isabella Teck, Judith L. MacManus-Driscoll

**Affiliations:** Department of Materials Science and Metallurgy, University of Cambridge, 27 Charles Babbage Road, Cambridge, CB3 0FS UK

**Keywords:** Artificial intelligence, Electronic material, Emergent phenomena, Ferroelectric, Nanoelectronics, Neuromorphic, Spintronic, Thin film

## Abstract

This prospective and performance summary provides a view on the state of the art of emerging non-volatile memory (eNVM) in the semiconductor industry. The overarching aim is to inform academic researchers of the status of these technologies in industry, so as to help direct the right academic research questions for future materials and device development. eNVM already have a strong foothold in the semiconductor industry with the main target of replacing embedded flash memory, and soon possibly DRAM and SRAM, i.e. replacing conventional memory. Magnetic and resistive memory are the current frontrunners among eNVM for embedded flash replacement and they are very advanced in this, which poses high demands on future academic research for eNVM for this purpose. Phase-change and ferroelectric memory are less available as commercially available products. The use of eNVM for new forms of artificial intelligence hardware is a much more open field for future academic research.

**Graphical abstract:**

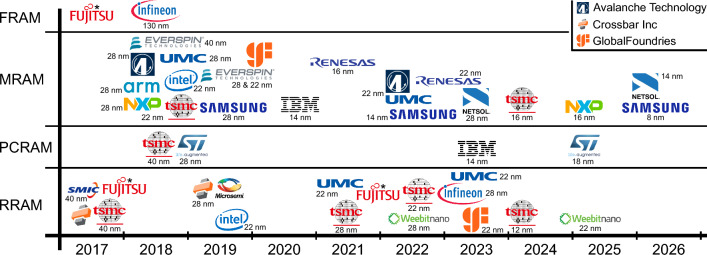

## Introduction

Emerging non-volatile memory (eNVM) is arguably one of the most intensively studied areas in semiconductor and nanoelectronics research and it stretches across subjects from materials design to applied physics to solid-state chemistry to electrical engineering to algorithm development. Naturally, at each stage and in each subject, the driving questions and research motivations differ, e.g. from fundamental conceptual understanding all the way to product development. Regardless of the underlying motivation, solving challenges of future data storage and artificial intelligence hardware is critical and is a prominent motivator in most research papers in the field.

In this prospective, based on publicly available information, we summarise existing company implementations of and corporate research efforts on eNVM technologies. Whilst a large number of academic reviews are available on all aspects of eNVM, amongst them there seems to be only the occasional look at corporate efforts in these areas.^[1]^ As eNVM research is ultimately targeted at end user products, a focus on industry efforts can thus add very valuable motivation and direction for future research in the field.

In a later section, “Company details,” we will provide details of commercially available technology by a number of companies active in the area of eNVM. Before delving into these details, we provide our conclusions derived from them as an executive summary and use it to derive recommendations for future eNVM research.

## Executive summary and research recommendations

**Figure 1:**
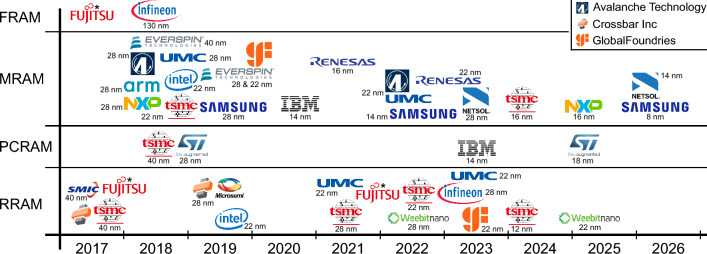
Timeline of recent technology demonstrations of eNVM with respective CMOS nodes. Sources for the different demonstrations can be found in the company details sections. Note that we only included companies with confirmed and active consumer products (notably, Intel/Micron 3D XPoint is not included, as it was discontinued), but for those, we included both products and research papers. *For Fujitsu, information about CMOS node integration could not be found. 2022 was one of their larger RRAM product launches, another was at least as early as 2016. For FRAM, 2022 was a major release, but their FRAM mass production started already in 1999. The top right inset clarifies companies where the name is not evident from the logo.

This review focusses on the four most advanced eNVM technologies; ferroelectric (FRAM or FeRAM), phase-change (PCRAM), resistive (RRAM), and magnetic (MRAM) memory. The lead of these four over other potential eNVM technologies is evident based on academic research efforts, their increasing compatibility with standard large-scale complementary metal-oxide-semiconductor (CMOS) fabrication, and consequently with their presence in consumer products or at corporate development stages. A timeline of how some of the major semiconductor companies have integrated these four eNVM with increasingly advanced CMOS nodes is provided in Fig. 1.

Whilst the acronym RAM—random access memory—actually describes an access architecture rather than a cell-level functionality, it has become customary to refer to the four mentioned technologies as FRAM (or FeRAM), PCRAM, RRAM (or ReRAM), and MRAM, even when referring to the technology on a single-cell level or as a concept, where the RAM architecture is not given. We will keep with this convention in this article for readability. Note that RRAM will include both oxygen-vacancy-based cells as well as metallic conductive bridge cells (CBRAM), PCRAM will also include selector-only memory (SOM) cells, and MRAM will typically be further specified as STT-MRAM, i.e. spin-transfer torque MRAM, or SOT-MRAM—spin-orbit torque MRAM. Furthermore, we will keep the company implementation details to a ‘cell’ level, i.e. looking at the typical figures of merit at that level, rather than discussing circuit-level aspects, such as memory density or bandwidth. This is because with this review, we want to provide benchmarks and inform future research for materials and device researchers, whilst circuit-level research is a very different area.

With details about various companies provided in the later text, it becomes clear that various eNVM are already available as consumer products at a large scale and at very advanced CMOS nodes down to 22 nm, with even smaller nodes at qualification and development stages. This is summarised in Fig. 1. The vast majority of implementations is for embedded memory, to replace embedded flash, but a few standalone eNVM products are available, too, and DRAM and SRAM replacements are picking up speed in development. Figure 1 summarises this solid foothold of eNVM in the semiconductor industry for conventional memory replacement, where by ‘conventional’ we mean using eNVM to provide and/or replace the same functionality as flash, DRAM, and SRAM. Amongst the four discussed technologies, RRAM and MRAM are more developed and available than FRAM and PCRAM, and unsurprisingly, the most advanced eNVM implementations come from major semiconductor companies, such as Samsung and TSMC.

Despite this advanced level of development of eNVM, it is still a common occurrence to see new publications in the field praising new-found “exceptional” or “highly promising” performance, when in reality, there are obvious gaps between academia and industry in the field of eNVM.^[2,3]^ For example, it is not fruitful to demonstrate a few hundred cycles of resistance switching in a novel exotic material and argue that this is promising for future memory applications. This is because major semiconductor companies have already reached advanced CMOS processing nodes with consumer-grade performance and many orders of magnitude cycling endurance. For memory applications, new research will have to improve where the current technology is lacking, for example, by demonstrating a reliable way to increase the number of switching cycles beyond what industry can already deliver. Alternatively, if in the example of cycling endurance, a new demonstration cannot compete with existing technology, the underlying research may still be valuable to the field if it helps to understand fundamental transport mechanisms or address more exploratory fields, such as neuromorphic computing or bioelectronics. Similar arguments apply to all other common eNVM metrics. Thus, future research in the eNVM area needs to answer the right questions and always be geared towards a clear technological or scientific goal. For conventional memory replacement, production cost is a factor as important as performance,^[2]^ as the market entry and exit of the Optane/3D XPoint memory demonstrates. Its performance was excellent, but the economies of scale did not make it profitable.

Besides the abovementioned conventional use of eNVM, they are under investigation for neuromorphic computing and new artificial intelligence hardware, and this field is much less advanced compared with conventional memory replacement with few to no commercially available products available, but are picking up speed. The area of emerging AI hardware is wide open as it does not seek to replace existing technology, but to create completely new forms of computing hardware. Consequently, there is much more room for future development than in conventional storage replacement.

## eNVM functionality basics

**Figure 2:**
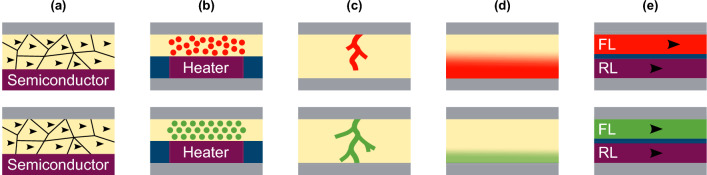
Schematic illustrations of the working principles of (a) FRAM, (b) PCRAM, (c) filamentary RRAM, (d) area-dependent RRAM, and (e) MRAM. Grey indicates electrodes and pale yellow indicates the memory material. For each, the top schematic represents the high resistance state, and the bottom represents the low-resistance state. The arrows in (a) indicate the ferroelectric polarisation orientation. The dots in (b) indicate the amorphous and crystalline phase of the material. The gradient in (d) indicates the height and width of a Schottky barrier. The arrows in (e) indicate the spin orientation and FL and RL stand for free layer and reference layer, respectively.

As a reminder and to provide physical context for the later company detail examples, we will summarise the basic working principles of the four key types of eNVM technologies. As there are scores of dedicated reviews available on each of the approaches, we will keep this very brief and superficial. Schematic illustrations are provided in Fig. 2.

FRAM relies on the reversible bi-stable spontaneous polarisation of a ferroelectric or anti-ferroelectric material. If a ferroelectric insulator replaces the gate dielectric in a metal-oxide field-effect transistor, the ‘up’ and ‘down’ polarisation of the ferroelectric gate insulator will cause shifts of the transistor threshold voltage, as one polarisation direction accumulates carriers in the channel, whilst the other depletes them, and the difference in threshold voltage can be used to store information. Figure 2(a) shows the gate/channel section of such a FeFET. Alternatively, the controllable polarisation can be used in a ferroelectric capacitor, where different directions of polarisation will cause differently strong read-out signals of the memory cell.

PCRAM makes use of the different conductivities of the crystalline or amorphous phase of a material, indicated in Fig. 2(b). Information can thus be stored in PCRAM by crystallising or amorphising the memory material and the resulting cell resistance contains the stored information.

For RRAM, the information is also stored in the resistance of a memory cell, but different from PCRAM, this is not achieved by a ‘bulk’ phase change. Instead, in most RRAM realisations, a reversible filamentary (oxygen vacancies or metal cations) breakdown is induced in the otherwise insulating memory material, as illustrated in Fig. 2(c). Alternatively, the oxygen concentration near one of the electrodes is changed, which changes the electronic energy barrier at this interface and leads to a resistance change in this way. This is illustrated in Fig. 2(d).

An STT-MRAM cell consists of a magnetic reference layer and a ‘free’ magnetic layer, separated by a magnetic tunnel junction (MTJ). When a current passes through the reference layer, its electrons become spin-polarised. When passing through the MTJ into the free layer, the polarised spins of a sufficiently large current can orient the magnetisation of the free layer, thus storing information. Inversely, a current passing first through the free layer can randomise its spins. The parallel or anti-parallel spin orientation in the two layers, illustrated in Fig. 2(e), will lead to larger or smaller read currents, respectively. In an SOT-MRAM cell, the write current does not pass through the full stack, but only through a metal lead adjacent to the free layer, and the spin transfer occurs via the spin Hall effect.

For conventional memory applications, the important figures of merit are well-known. Amongst others, they include cycling endurance, data retention, access (write and read) speed, switching voltages, currents, energies, and size. Table I provides a high-level summary of the strengths and weaknesses of the four discussed eNVM typically discussed in review papers^[1]^ and Table II presents some of the top performance parameters from the company details summarised below. Comparison of the two tables shows that whilst some of the commonly assumed weaknesses are reflected in commercially available technologies, company development has already managed to overcome others. This demonstrates clearly that ‘it can be done.’ Often, the solution will be highly specific to a company and product, but for each company, this is all it takes for mass production. In Table I, the generalised strengths and weaknesses are adopted from Ref. 1 as a representative example, with radiation hardness concluded from Ref. 4. In Table II, the values for DRAM and SRAM integration nodes are taken from Ref. 5 and Ref. 6, respectively, and the other flash, DRAM, and SRAM metrics from Ref. 7 and references therein. Note that whilst NAND flash scales down to the 1X-nm nodes, e.g. 16 nm already in 2013,^[8]^ NOR flash, which is used for embedded flash, faces major difficulties to scale beyond 28 nm.^[9]^

**Table I:** High-level summary of advantages and disadvantages typically propagated in eNVM reviews, such as Ref. 1.

Feature	FRAM	PCRAM	RRAM	MRAM
Endurance	High	Moderate	Moderate	Very high
Data retention	Moderate	Moderate	High	Moderate
Write speed	Fast	Moderate	Fast	Fast
Read speed	Fast	Moderate	Fast	Fast
Power consumption	Low	High	Low	Moderate
Density	Moderate	High	High	Moderate
Cost per bit	Moderate	Moderate	Low	High
Fabrication complexity	Moderate	Moderate	Low	High
Radiation hardness	High	High	High	High

They are generally reflected in the demonstrated performance in Table II, although some of the best performances indicate that many of the gaps between technologies can be closed. Radiation hardness is taken from Ref. 4.

**Table II:** Summary of top metrics from the detailed company information in the text.

Metric	FRAM	PCRAM	RRAM	STT-MRAM	NOR flash	DRAM	SRAM
Endurance	10^15^ (Infineon, Texas Instruments)	>10^7^ (IBM/Macronix, SK hynix SOM*)	10^12^ (Samsung)	10^16^ (Avalanche)	10^4^–10^6^	10^16^	10^16^
Retention without power	121 years at 85℃, 11K h at 125℃ (Infineon)	>10 years at 150℃ (STMicro)	10 years at 105℃ (TSMC)	20 years at 150℃ (TSMC), 10^6^ years at 65℃ (Renesas)	>10 years	100 µs	None
Write speed	2 ns (Intel)	≤20 ns (SK hynix SOM*)	5/1 ns set/reset (Sony)	1.5 ns (TDK)	0.1–1 ms	∼10 ns	∼1 ns
Read speed	2 ns (Intel)	≤30 ns (SK hynix SOM*)	<5 ns (Intel)	4 ns (TDK)	∼10 ns	∼10 ns	∼1 ns
Switching voltages	0.5 V (SK hynix)	<3 V (STMicro)	<1–1.5 V (Intel)	0.8–2.0 V (Avalanche)	>5 V	<1 V	<1 V
Cell write energy	<100 fJ (Intel, projected)	<2.63/1.5–9 pJ set/reset (Samsung/KAIST)	5 fJ (Samsung/POSTECH)	0.7 pJ (Toshiba)	∼100 pJ	∼10 fJ	∼fJ
Integration node	45 nm line pitch (Micron)	14 nm (IBM)	12 nm CMOS (TSMC)	14 nm CMOS (IBM)	28 nm**	10 nm (1c) (SK hynix)	3 nm (TSMC)

These metrics come from both product information and company research papers, to provide values ‘to beat’ by future research. The companies behind each achievement are included in parentheses and the corresponding references can be found in the “Company details” section below. Comma separations indicate that various companies have demonstrated the performance; slash separation indicates collaborations. The flash, DRAM, and SRAM values are taken from Refs. 5–7. Note that other companies besides SK hynix and TSMC have achieved similarly advanced DRAM & SRAM notes; these two are just examples.

*SOM: selector-only memory.

**Whilst NAND flash has been demonstrated down to the 1X-nm node, NOR flash (for embedded memory) generally stops at 28 nm.

## eNVM intellectual property

**Figure 3:**
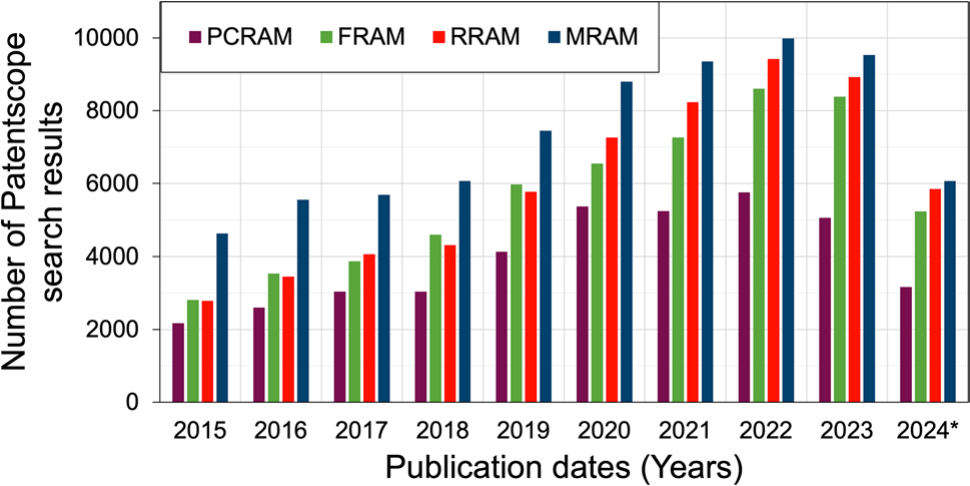
World Intellectual Property Organisation Patentscope search results from 2015 to July 2024 for the four included eNVM. The number of IP applications has been steadily increasing over the years. In line with Fig. 1, RRAM and MRAM are the frontrunner technologies, but the others are not far behind. Patentscope search settings were office = all, languages = en, stemming = FALSE, single family = FALSE, and including NPL = FALSE. Search terms for the individual eNVM technologies were as follows: ALLTXT:(“phase change random access memory”) or (“phase change RAM”), ALLTXT:(“ferroelectric RAM”) or (“ferroelectric random access memory”), ALLTXT:(“resistive random access memory”) or (“resistive RAM”), and ALLTXT:(“magnetoresistive RAM”) or (“magnetic RAM”) or (“magnetoresistive random access memory”) or (“magnetic random access memory”).

Before finally delving into some company technology details, we provide a high-level intellectual property (IP) overview by summarising search results from the World Intellectual Property Organisation (WIPO) database Patentscope. The results are presented in Fig. 3 and clearly show the constant increase in applied-for IP over the years for the four discussed eNVM technologies. In line with Fig. 1, RRAM and MRAM are the current frontrunner technologies, but the other two are not far behind.

As the results obviously depend on the search parameters, Fig. 3 should be viewed as a trend, not an accurate quantification. With the detailed search parameters provided in the figure caption, we used no abbreviations other than RAM (e.g. RRAM was written out as “resistive RAM” or “resistive random access memory”) to avoid abbreviations in text being associated with completely unrelated topics, e.g. PCRAM being a common abbreviation for “physical climate risk assessment methodology”.

## Company details

In the final section before the summary and outlook, we summarise the information we could obtain from company homepages, press releases, industry research papers, datasheets, and tech blogs. As such, this review part is a bit different from typical academic review papers in that many of its references are not academic papers, but web page accesses, press releases, and datasheets. To ensure accountability, we provide snapshots or copies of all cited open-access sources in a repository entry linked to this article (doi in section ‘Reference availability’). As mentioned in the introduction, we limited performance parameters to the cell level rather than circuit level to provide benchmarks for materials and device researchers. Due to the limited length of the format, we had to make a selection of companies to include, and we tried to do so based on the impact of their recent contributions to the field, or how prominently they were seen in the field. Naturally, this is subjective to a certain extent. As is further the nature of a review, we may easily have missed important information, and we apologise for any important omissions. Further due to restrictions in the number of references, there was no room to include references for company or IP acquisitions, so we focused on providing references for important performance data. As a disclaimer, this work does not contain any sponsored content.

**4DS Memory Limited**—Different from most companies working on RRAM, whose technologies are typically filament-based, 4DS use an area-dependent type of switching. Their devices are based on oxygen ion movement in a praseodymium calcium manganese oxide (PCMO) perovskite layer. Megabit arrays with 60 nm cells have been demonstrated as well as 40 nm cell feasibility. The technology is advertised with switching endurances of up to 10^9^ cycles and write speeds of 4.7 ns.^[10]^ The company is working towards licensing their approach as a fully back-end-of-the-line-compatible technology and do not currently fabricate devices.

**Adesto Technologies**—Adesto produced conductive bridge random-access memory (CBRAM) devices, but was acquired by Dialog Semiconductor in 2020, which in turn became a subsidiary of Renesas in 2021. Renesas then sold their CBRAM IP to GlobalFoundries. Performance details are provided in the GlobalFoundries section.

**Advanced RISC Machines (ARM)**—In 2020, ARM spun out Cerfe Labs to commercialise their research on correlated electron memory (CeRAM), based on metal–insulator phase transitions in transition metal oxides. Materials under investigation include doped metal oxide films such as NiO and undoped vanadium oxides.^[11]^

In addition to CeRAM, ARM has shown an interest in STT-MRAM. In collaboration with Samsung, a 28 nm MRAM macro was presented at VLSI 2020 with 1 V/1.8 V power supply, 33 ns read access time, 1.2 pJ/bit read energy, 10^6^ cycles of write endurance, state retention of 10 years at 105℃, and operative between −40 and 105℃.^[12]^ However, the focus of the presentation was the design of the read circuitry, which is closer to ARM’s core business of circuit design.

**Avalanche Technology**—Founded in 2006, their focus is on magnetic memory in the form of discrete and embedded STT-MRAM as well as system-on-chip architectures for Internet of Things (IoT), industrial, and space applications, and their MRAM has been integrated down to 22 nm. Typical performance parameters for the 28 nm product lines include operating voltages of 0.8–2 V (lowest reported amongst companies in this summary), a temperature range of −40 to 150℃, write endurances of 10^9^–10^16^ cycles (highest reported amongst companies in this summary), minimum data retention of 20 years at 85℃, and read/write times of down to 10/20 ns.^[13]^ The 22 nm node was announced in 2022 in collaboration with UMC and offers similar performance as the previous node, such as >10^14^ write cycle endurance, 10^3^ years retention at 85℃, and operating temperatures of −40 to 125℃.^[14]^

**Crossbar Inc.**—Founded in 2010, they commercialised RRAM with a metallic filament in a silicon-based host material for various applications such as IoT, AI, data centres, mobile computing, and security applications in the form of secure keys embedded in semiconductors by making use of the inherent randomness in RRAM devices. Some key characteristics are an operational temperature range of −40 to 125℃, >10^6^ write cycles, read/write latency of 20 ns/12 µs, data retention of 10 years at 85℃, and 3D stackability resulting in advertised scalability <10 nm.^[15]^ Overall, it seems like Crossbar are looking to license their IP rather than manufacture themselves, and licensing is advertised to be ready for 2X nm and 1X nm nodes. In 2018, for example, Crossbar licensed their 28 nm core RRAM technology to Microsemi, and this collaboration led to the 1X nm research.^[16]^ However, Microsemi was bought by Microchip Technologies, who do not seem to pursue the technology. More details in the Microchip section.

**Cypress Semiconductors**—Before being acquired by Infineon in 2020, Cypress developed FRAM, which can now be found in Infineon products. More details in the Infineon section.

**Dialog Semiconductors**—Working on CBRAM, they licensed their IP to GlobalFoundries first in 2020. Dialog was acquired by Renesas in 2021, from which GlobalFoundries bought out the CBRAM IP in 2023.

**EverSpin Technologies**—Founded in 2008, they are one of the most successful MRAM startups. For example, in 2024 it was announced that Everspin’s STT-MRAM will be used in IBM’s FlashCore modules. Some of the MRAM specifications are operating temperatures of 0 to 85℃, 667 MHz clock frequency, data retention of 3 months at 70℃, and a cycling endurance of 10^10^.^[17]^ EverSpin produces STT-MRAM in the 180 nm, 130 nm, and 90 nm nodes themselves and since 2014, they have been working with GlobalFoundries to develop high-volume production of MRAM in the 40 nm, 28 nm, and 22 nm nodes and in 2020, the collaboration was extended to the 12 nm node.^[18]^

**Ferroelectric Memory Company (FMC)**—Spun out from TU Dresden/NaMLab in 2016, they work on embedded non-volatile FRAM based on hafnium oxide. Whilst products or licensing do not yet seem to be ready, published performance includes operating temperatures from −190 to 200℃, switching endurance of >10^9^ cycles, >1000 h data retention at 125℃, and a switching speed in the 10 ns range.^[19]^

**Fujitsu Semiconductor Memory Solution Ltd.**—Besides a strong interest in FRAM and RRAM, Fujitsu are also actively working on carbon-nanotube-based memory (NRAM). The latter technology was licensed from Nantero, and whilst not yet listed as a product, Fujitsu have demonstrated an NRAM macro on 55 nm CMOS with set/reset voltages of 2.5/3.5 V, speeds of 200/100 ns on an array level, 0.5 ns switching of individual cells, read voltage of 0.5 V, 10^5^ h extrapolated retention at 150℃, and a write endurance of 10^6^ cycles.^[20]^

For FRAM, Fujitsu provide a variety of products for serial and parallel memory with write endurances varying between 10^10^ and 10^14^ cycles, operating temperatures between −40 and 125℃, supply voltages of 1.7–3.6 V, and operation frequencies of up to 108 MHz.^[21]^ In a 2023 RRAM white paper, Fujitsu highlighted RRAM read currents of 0.15–1 mA at 5 MHz operating speed, with supply voltages between 1.6 and 3.6 V, a write endurance of 5 × 10^5^ cycles, and unlimited read endurance.^[22]^

**GlobalFoundries (GF)**—In a collaboration with EverSpin, they have successfully embedded MRAM down to the 22 nm node, with 12 nm in development,^[23]^ and based on the IP which GF bought from Renesas, 22 and 12 nm CBRAM are at the development stage as of 2023.^[23]^ Further efforts on 22 nm are ongoing in a collaboration with Weebit Nano.^[24]^ Demonstrated performance parameters can be found in the EverSpin and Weebit Nano sections.

**Headway Technologies Inc.**—As a part of the TDK corporation, they are focused on STT-MRAM development and already in 2014, they demonstrated write pulse lengths down to 1.5 ns, read pulse lengths down to 4 ns, estimated data retention up to 125℃ for 10 years, and cycling endurance >10^4^.^[25]^

**International Business Machines Corporation (IBM)**—Whilst their business model has changed over the years, IBM has stood out amongst companies for many years in being very public about their IBM Research labs. In their products, IBM use MRAM as buffers in their FlashCore modules (similar to SSD) and recently announced that they will use Everspin technology for the new generation products, despite having demonstrated in-house STT-MRAM in a 14 nm node (smallest amongst companies in this summary) in 2020.^[26]^ The integrated MTJ cells of the macro consisted of CoFeB-based free layers, MgO tunnel barriers, and an unspecified synthetic anti-ferromagnet reference bottom layer. Single MTJs in the macro could be switched >10^10^ times and with speeds down to 4 ns, but data retention was only demonstrated for ≤1 min, clearly positioning this technology as a DRAM/SRAM replacement rather than non-volatile memory. At VLSI 2024, in collaboration with Samsung, they followed up with down to 2 ns switching.^[27]^

In addition to MRAM, IBM are also looking into PCRAM, RRAM, and FRAM. All three types of memory are being investigated for in-memory computing^[28]^ and PCRAM is also being investigated for crossbar memory. Recent PCRAM demonstrations, together with Macronix, are based on Ge_2_Sb_2_Te_5_ (GST-225) with an unspecified dopant (oxygen + ‘A’) and In-doped AsSeGe selectors. They achieve 10^7^ chip-level switching cycles (highest amongst companies in this summary, shared with SK hynix) with 400 ns set pulses, 50 ns reset pulses, and 800∼1400 µA reset currents, as well as negligible drift after one-day baking at 85℃.^[29]^ IBM has used PCRAM for deep neural network inference, where the PCRAM was integrated at the back end of the line on top of a 14 nm CMOS process,^[30]^ which is the smallest PCRAM integration among companies in this summary. Further in collaboration with Macronix, and presented at VLSI 2024, selector-only memory is being explored,^[31]^ but the conference proceedings were not yet available at the time of writing this article. Some of the RRAM demonstrations for in-memory computing seem to combine RRAM and PCRAM.^[32]^ Example metrics for FRAM in-memory computing include >10^8^ switching cycles with ±2 V at 100 KHz based on a TiN/HfZrO_4_/TiO_*x*_/TiN/SiO_2_ stack.^[33]^

**Infineon**—Both FRAM and RRAM can already be found in their products; FRAM as the standalone EXCELON™ and radiation-hard memory and RRAM integrated in their automotive AURIX™ microcontrollers (in collaboration with TSMC) and edge machine learning microprocessors. Based on the documentation on their homepage (datasheets in 2024 still with Cypress logo), the FRAM technology is strongly based on technology developed by Cypress Semiconductor Corporation, which Infineon acquired in 2020. Infineon’s FRAM operates at supply voltages between 1.71 and 3.6 V, temperatures between −55 and 125℃, sports an endurance of 10^15^ switching cycles, which, together with the performance by Texas Instruments, is the highest value we found for this summary (10^13^ cycles for rad-hard), data retention of 121 years at 85℃ and 11 K hours at 125℃, which is the highest we found for FRAM for this summary, and for rad-hard applications, it can sustain a total radiation of 150 Krad.^[34]^ There is less information available on the RRAM, but it is qualified at least for automotive applications, which entails 125K switching cycles, 1000 h data retention at 175℃, and operating temperatures between −40 and 160℃.^[35]^

About 20 years ago, Infineon was also working on MRAM, together with IBM, but there do not seem to be any more updates since then. Instead, documents appeared which indicate that Infineon may have abandoned MRAM in favour of Cypress’ nvSRAM, which is essentially a conventional SRAM cell integrated with non-volatile silicon–oxide–nitride–oxide–silicon (SONOS) flash-like transistors for data storage if the power drops.^[36]^

**Intel**—As one of the most advanced technology drivers in the world, together with Micron, Intel introduced their phase-change-based 3D XPoint memory ‘Optane’, which they unfortunately had to discontinue in 2022, as the economies of scale did not take off. Already whilst Optane was still running, Intel introduced MRAM and RRAM in their 22 nm node at IEDM 2018 and VLSI 2019, respectively.

The MRAM introduction macro was based on an MgO MTJ and a CoFeB-free layer and featured 10 years retention at 200℃ combined with >10^6^ cycles of switching endurance, a tunnelling magnetoresistance ratio of >180%, write pulse speeds of 80 ns (20 ns demonstrated, but with increasing errors), and read speeds of 10 ns.^[37]^ A presentation of the same technology at ISSCC 2019 further specified a read disturb endurance of >10^12^, read sense times of 4 ns at 0.9 V and 8 ns at 0.6 V, and operating temperatures between −40 and 105℃.^[38]^

The 22 nm RRAM technology is based on a tantalum-based, but further unspecified, oxygen exchange layer on top of a TaO_*x*_ switching layer and a noble metal electrode. Typical values for the high and low resistance states were provided as 3–7 KΩ and >30 KΩ, respectively, with typical forming voltages of 1–1.5 V, which are the lowest values amongst companies in this summary, cycling endurance of 10^4^ cycles, and 10 years data retention at 85℃.^[39]^ Just as with the MRAM introduction, further details were provided in a 2019 ISSCC presentation as −40 to 125℃ operating temperature, <5 ns sense time (fastest reported amongst companies in this summary) at 0.7 V and <10 ns at 0.5 V, leading to <1 pJ read energy, whilst write times were 10 µs.^[40]^

In addition to MRAM and RRAM, Intel also seems to have a strong interest in FRAM for next-generation DRAM. At IEDM 2022, they demonstrated 1-transistor-1-resistor (1T1R) FRAM based on hafnium zirconium oxide (HZO) capacitors with TiN electrodes.^[41]^ The results demonstrated −1.8 to 1 V switching voltages with pulse widths as low as 2 ns (fastest reported amongst companies in this summary), and 10^12^ switching cycles at 85℃ with sense voltages of 0.5 V. State retention only seemed to work for about an hour, though. Intel presented similar results based on anti-ferroelectric hafnia-based capacitors at VLSI 2024: bit and plate line write voltages of down to 1 and 1.3 V, respectively, read voltages down to 0.6 V, 10 years retention at “elevated temperatures”, and projected <100 fJ write/read cell energy, which is the lowest amongst companies in this summary.^[42]^

**IntrinSic Semiconductor Technologies**—As an RRAM startup from University College London, their company data is still classified, but a journal publication, which their technology is based on, reveals performance metrics of switching voltages of ±1 V, switching endurance of 10^7^ cycles, and state retention of 1 h at 260℃, all based on filamentary switching in amorphous silicon oxide.^[43]^

**Kioxia**—Formerly Toshiba Memory Corporation, they claim the invention of both flash memory and vertical flash memory. Whilst there does not seem to be any product information available on their homepage, based on their most recent conference publications, for potential future eNVM, Kioxia seem to be most interested in MRAM and FRAM. They presented a 14 nm (by electron beam lithography) MTJ based on a doped CuPt alloy “high retention layer” on top of a CoFeB “switching accelerator layer”, an MgO barrier, and an unspecified reference layer. Provided example performance includes >10 years retention at 90℃ and down to 5 ns write speed.^[44]^ Already in 2013, then still Toshiba, they presented an STT-MRAM macro with 250 MHz operating speed and bit write energy as low as 0.7 pJ, the lowest amongst companies in this summary, integrated in a 65 nm CMOS process.^[45]^ As an FRAM example, a Si channel hafnium oxide FeFET was presented with state retention of 10 years and a memory window of 2.3 V after 10^7^ switching cycles with ±6 V switching voltages.^[46]^

**Macronix**—Their most recent eNVM demonstrations include ferroelectric HZO^[47]^ (with National Taiwan University) and chalcogenide-based selector-only memory^[31]^ (with IBM) at VLSI 2024. The proceedings were not yet accessible at the time of this review. From the abstracts alone, the FE HZO was ca. 10 nm thick, integrated on TiN, and achieved 2*P*_r_ = 56 µC/cm^2^ after >10^12^ switching cycles. No performance data were available from the abstract on selector-only memory, which is based on CuGeSe.

**Microchip Technology Inc.**—Despite Microchip’s acquisition of Microsemi in 2018, who at the time had licensed Crossbar’s 28 nm RRAM technology, it seems that instead of pursuing any of the eNVM, Microchip adopted a more conventional approach to achieve a non-volatile SRAM cell by combining the typical 6-transistor SRAM cell with flash transistors to ‘save’ the SRAM data if the power supply drops too low.^[48]^

**Micron**—Unfortunately known for the ultimately unsuccessful attempt of introducing 3D XPoint with Intel, they since seem to have shifted their focus onto other technologies. Similar to SK hynix, they seem to have concluded that a single-chalcogenide selector-only-like memory may be more promising than the previous PCRAM approach. Unfortunately, the provided publication document is heavily obscured and/or defective, but it reveals that the prototype is based on a 20 nm integrated chalcogenide threshold switch, which can sustain at least >10^7^ switching cycles.^[49]^

In parallel, Micron presented a ferroelectrics-based DRAM-like NVM macro, which consists of dual-layer 1-transistor-1-capacitor (1T1C) cells with a 48 nm CMOS pitch,^[50]^ which is the smallest corporate FRAM integration amongst companies in this summary. The FE memory capacitor uses a 5.7-nm-thin doped orthorhombic HZO layer and can be cycled >10^12^ times with a 1.5 V operating voltage whilst maintaining 2*P*_r_ > 55 µC/cm^2^. Memory operation is demonstrated at temperatures between −40 and 95℃ and data retention is provided as >10 years at 55℃.

Both of these efforts seem to be very much at the research and exploratory stage, but could well indicate the new direction that Micron is taking after the hit they took with 3D XPoint.

**Nantero**—Different from the majority of RRAM technologies, Nantero use a carbon nanotube (CNT) ‘fabric’ as the active layer. Set and reset voltages ‘open’ and ‘close’ connections between adjacent CNTs to set the resistance. The technology has demonstrated hundreds of years of projected state retention at temperatures between −55 and 300℃, can be programmed with 5 ns pulses, and has already been scaled to a 15 nm cell size.^[51]^ The technology was also licensed to Fujitsu, who have demonstrated a macro on 55 nm CMOS, set/reset voltages of 2.5/3.5 V with speeds of 200/100 ns on an array level, 0.5 ns switching of individual cells, read voltage of 0.5 V, 10^5^ h extrapolated retention at 150℃, and a write endurance of 10^6^ cycles.^[20]^

**Netsol**—They mass-produce various flavours of standalone parallel and serial STT-MRAM based on Samsung’s 28 nm node with plans to scale to 14 nm in 2026.^[23]^ Performance parameters advertised on their homepage include operating voltages of 1.7–3.6 V, operating temperatures between −40 and 85℃, data retention of 10 years, unlimited read and 10^14^ cycles of write endurance, and up to 108 MHz operating frequency (for serial memory).^[52]^

**Nuvoton**—Several of their microcontrollers contain RRAM and this seems to be their main eNVM technology. They acquired Panasonic’s chip business in 2020 and it seems the RRAM technology came with it. Indeed, over the following years, research articles emerged in collaboration with the University of Tokyo about tantalum oxide RRAM for compute-in-memory studies, and the RRAM resembles the earlier Panasonic technology. Example metrics include a cycling endurance of 10^4^, switching voltages of 2.7/2 V, and a read voltage of 0.4 V.^[53]^

**NXP**—In 2018, they announced production of microcontrollers with Samsung’s 28 nm STT-MRAM and in 2023, they announced that they will deliver MRAM in a 16 nm FinFET architecture for automotive applications together with TSMC in early 2025.^[54]^ This seems by far the only publicly available information about NXP eNVM. 10^6^ cycles of endurance and 20 years data retention at 150℃ were provided as example metrics.^[54]^ More details in the TSMC section. Recent job openings indicate that NXP may be sampling RRAM in addition to MRAM.

**Panasonic/PSCS (Panasonic Semiconductor Solutions)**—They were the first company to mass-produce RRAM in 2013 and RRAM seemed to be their eNVM technology of choice. In 2015, in collaboration with imec, they presented a 40 nm RRAM macro with 10^5^ endurance cycles and 10 years retention at 85℃,^[55]^ whilst reporting unlocking the 28 nm node. Yet in 2020, Panasonic went back to reporting the same performance in their 40 nm node,^[56]^ which puts a question mark on their further progress. Panasonic’s early adoption of Symetrix FRAM in rail commuter cards in 2008 indicates an interest in that area, too, but little to no information seem publicly available. In 2020, Nuvoton acquired Panasonic’s chip unit, likely taking over the eNVM business.

**Renesas**—Since they sold their RRAM IP to GlobalFoundries in 2023, their eNVM technology of choice is STT-MRAM with target applications as embedded and small-scale standalone memory. Both 22 nm and 16 nm node macro implementations have been demonstrated and typical end user product performance (40 nm) includes extrapolated 10 years data retention at 105℃ (10^6^ years at 65℃, highest reported amongst companies in this summary, shared with Avalanche), write endurance of 10^14^ cycles, read and write cycle times of 35 ns, supply voltages of 2.7–3.6 V, and operating temperatures of −40 to 105℃.^[57]^ Whilst an end-of-life notice in 2023 indicates the discontinuation of many of Renesas’ MRAM products in 2024,^[58]^ further product development is still very much continuing.^[59]^

**Samsung**—Their main product to date is embedded STT-MRAM, and they seem to be amongst the most advanced positions for this technology. Samsung MRAM was first shipped through mass production in 2019 based on their 28 nm fully depleted silicon on insulator (FD-SOI) platform. At IEDM 2022, the 28 nm MRAM was also presented as a standalone technology with operating voltages of 1.8/3.3 V, operating temperatures of −40 to 85℃, 40/160 ns read/write speed, a switching endurance of 10^14^ cycles, although at −25℃, and 10 years data retention at 89℃.^[60]^ In the same paper, a further improvement to an embedded 14 nm node was presented (−40 to 105℃ operating temperature, 15/50 ns read/write speed, 12 pJ/bit write energy), and the 14 nm automotive technology was presented at VLSI 2024 with a switching endurance of 10^12^ cycles, 100 ns write speed, and 10 years data retention at 150℃.^[61]^ Also at VLSI 2024, in collaboration with IBM, <5 ns switching STT-MRAM was presented with high *H*_c_ > 8 KOe and high *E*_b_ > 80 *k*_B_*T*.^[27]^ Samsung 8 nm MRAM is planned by 2026 and 5 nm by 2027.^[62]^ Samsung also demonstrated the use of MRAM for in-memory computing, e.g. in Nature 2022.^[63]^

As their second eNVM technology of choice, in collaboration with various partners, Samsung is investigating FRAM, as reflected most recently, for example, by their VLSI 2024^[64]^ (with KAIST) presentations, or by more foundational work with the University of Cambridge, UK.^[65]^ The targeted applications include NAND flash replacement for up to 1000-layer vertical NAND^[64]^ and in-memory computing^[66]^ (e.g. with Sungkyunkwan University). For NAND flash replacement, a memory window as high as 10.5 V was demonstrated, although with switching voltages as high as +17/−15 V.^[64]^

Despite highly promising research results on tantalum oxide RRAM as early as 2011,^[67]^ with a switching endurance of 10^12^ cycles, the highest value we are aware of for RRAM amongst companies in this summary, cycles and switching speeds as fast as 10 ns, for many years, Samsung proclaimed low interest in RRAM and also PCRAM, other than fabrication for other manufacturers, such as STMicro. But recent collaborative publications might indicate a renewed or at least quietly persistent interest. At VLSI 2024, for RRAM, multi-bit (4 bits) capability, switching speed ~50 µs, stable endurance >10^7^ cycles, and low switching energy consumption of 5 fJ (lowest reported amongst companies in this summary) were demonstrated alongside switching voltages <5 V in a POSTECH/Samsung collaboration.^[68]^ For PCRAM, in Nature, also in 2024, and in a collaboration with KAIST, a SiTe_*x*_ nano-filament PCRAM cell was demonstrated with set/reset currents of approx. 5/10–60 µA for switching voltages <|3.5| V, set/reset speeds of down to 150/<20 ns, set/reset energies of down to <2.63/1.5–9 pJ, the lowest value we are aware of amongst companies in this summary, on/off ratio >100, and a switching endurance >10^5^ cycles.^[69]^ Note that not all values were achieved together (e.g. at 150 ns, the memory window was 10 instead of 100).

**SK Hynix**—Based on recent research papers, they seem to be looking into all three of RRAM, FRAM, and MRAM, but their favoured technology seems to be different from almost all other major companies—selector-only memory (SOM). The latter is especially in contrast with earlier SK hynix efforts on PCRAM, from which they concluded SOM to be the better alternative. Curiously, given that they are one of the largest memory providers in the world, and different from their competitors, they do not yet seem to have set up mass production of any eNVM product.

SK hynix seem to be interested in RRAM mostly for in-memory computing. For this, they use hafnium-oxide-based devices with a thermal enhancement and a reservoir layer on top, and a buffer layer underneath the hafnium oxide to achieve stable multi-level filamentary switching. The additional layers are of undisclosed materials and performance optimisation is already at the macro and programming stage.^[70]^ For FRAM, the interest is spurred both by in-memory computing^[71]^ and potential DRAM^[72]^ or flash^[73]^ replacement. As the ferroelectric layer, HZO or Si-doped hafnium oxide is being used. Most recently, they demonstrated 3-nm-thin ferroelectric HZO capacitors, annealed at temperatures below 400℃, with 2*P*_r_ ≈ 55 µC/cm^2^ at an operating voltage of 0.5 V, which is the lowest voltage amongst companies in this summary.^[74]^ For in-memory computing and NAND flash demonstrations, FeFETs were used and for DRAM, capacitors. As is typical for company presentations, much of the data is normalised or otherwise obscured, but for FeFETs or capacitors, at least 4 bit/cell operation was demonstrated, for FeFETs endurance up to 3K cycles, and for capacitors up to 10^9^.

For SK hynix PCRAM, despite the promising demonstration of a 256 Gb macro with read/write latencies of 385/100 ns, programming currents <100 µA, cycling endurance of 10^6^, and non-volatility, the conclusion seems to be that a selector-only configuration is more promising than a 1-selector-1-memory (1S1M) selector plus phase-change material, because the conventional PCRAM faces challenges of scaling beyond 20 nm and thermal disturbance. This is especially noteworthy given that PCRAM is generally considered strong in scaling, cp. Table I. Indeed, the parallel demonstration of a selector-only crosspoint technology showcases improved values of read/write latencies of ≤30/20 ns (fastest for PCRAM amongst companies in this summary), cycling endurance of >10^7^ (highest amongst companies in this summary, shared with IBM), no thermal disturbance, and better scaling beyond 20 nm, albeit with considerable state drift during a measured 10^5^ s interval.^[75]^ Based on this research, the SOM technology is promoted most strongly publicly.

Finally, first in collaboration with Toshiba^[76]^ and then with Kioxia,^[77]^ SK Hynix demonstrated STT-MRAM macros. Between the two demonstrations, read/write latencies ≤30 ns, operating voltages as low as 1.1–1.7 V, and a measured endurance >10^6^ were achieved at dimensions of 20–90 nm. The target seems to be mainly DRAM replacement.

**Sony**—Whilst traditionally not a main player in the memory market, they have been amongst the early adopters of emerging memory technologies (e.g. FRAM in Playstation 2 memory card as early as 2000). In 2019/20, it seemed like Sony was close to developing a crosspoint RRAM, although the respective presentation^[78]^ featured the same results as their 2007 IEDM paper.^[79]^ There, the device performance was top notch already, with set/reset pulse widths of 5/1 ns (fastest amongst companies in this summary), set/reset currents of 110/125 µA, set/reset voltages of 3/−1.7 V, a read voltage of 0.1 V, switching endurance nearing 10^8^, state retention >10^3^ s, and multi-level capability. The material stack consisted of a Cu-Te-based top electrode on top of a GdO_*x*_ active layer with Cu diffusing into it in a CBRAM fashion. Since the 2019 announcement, there does not seem to have been much information, however. Instead, there are several recent research papers on ferroelectric and magnetic memory.

The FRAM work was carried out in collaboration with NaMLab for memory applications^[80]^ and with imec for in-memory computing.^[81]^ The former showed 10^12^ endurance cycles and extrapolated 10 years data retention at 85℃ for a 1T1C configuration with an operating voltage of 2.4 V. The capacitor for the 1T1C and the gate oxide for the 1T1R in-memory computing configuration were both based on HZO. At VLSI 2024, Sony researchers presented an HZO FRAM SRAM macro, again in collaboration with NaMLab, and with the Fraunhofer IPMS.^[82]^ For the magnetic memory macro, an MTJ was integrated with a 6T SRAM cell and showed non-volatile RAM functionality with operating voltages ≤0.72 V at speeds of 8–20 ns.^[83]^ Both the FRAM and MRAM memories are targeted at SRAM or DRAM replacement.

**STMicro (STMicroelectronics NV)**—Whilst individual STMicro products contain RRAM cells, their favoured technology seems to be PCRAM as per their homepage information,^[84]^ which reveals that the memory cell consists of germanium antimony telluride (GST) and will be integrated with 18 nm FD-SOI, capable of operating at 3 V (lowest value amongst companies in this summary) at temperatures of up to 165℃. Whilst 18 nm product lines are to be released later in 2024 or in 2025, the 2018 IEDM paper on the 28 nm technology showcased that >10^6^ switching cycles and extrapolated 10 years retention at 150℃ (highest value amongst companies in this summary) were achieved, too, with read currents on the order of 10 µA at read voltages <1 V.^[85]^ Main target applications are industrial, automotive, and aerospace, where the latter two had already been achieved with the initial 28 nm process,^[84]^ and fabrication seems to be carried out by Samsung on an STMicro/Samsung joint FD-SOI process.^[86]^

**TDK Corporation**—This corporation with a huge variety of products demonstrated a first memory chip based on STT-MRAM in 2014 through their subsidiary TDK Headways, with specifications of write pulse lengths down to 1.5 ns, read pulse lengths down to 4 ns, both of which are the fastest we found for MRAM amongst companies in this summary, estimated data retention up to 125℃ for 10 years, and cycling endurance >10^4^.^[25]^ Apart from this initial demonstration, there is little technical information available online, except for continuous patent filings in the area of STT-MRAM and SOT-MRAM, which seems to be their main interest. As recent as 2023, TDK confirmed that they are looking to apply their MRAM expertise to AI accelerators.^[87]^

**TetraMem**—Founded in 2018 as a spin-out from the Universities of Southern California and of Massachusetts Amherst, they are currently possibly the only company with a multi-level RRAM chip for AI acceleration, rather than using eNVM to replace existing memory. An underlying academic publication showcases quasi-analogue 2048 resistance levels with 10∼800 µA currents at a read voltage of 0.2 V, and at least 1000 s state retention without decay. The devices consist of a Pt bottom electrode, a Ti/Ta top electrode, and a HfO_2_/Al_2_O_3_ switching bilayer.^[88]^

**Texas Instruments**—According to the application notes and FAQs on their homepage, Texas Instruments have been using lead zirconate titanate (PZT) FRAM in their microcontrollers for more than 10 years.^[89]^ We could not find any information about efforts on other types of eNMV. With their long history in using FRAM, its specifications are provided as 1.5 V operating voltage, <50 s write time per cell, 10^15^ endurance cycles (highest number for FRAM amongst companies in this summary, shared with Infineon), 10 years retention at 85℃, and 100 years at 25℃.^[89]^ The PZT seems to be integrated as 70-nm-thick layers in a 130 nm process node. The main application targets seem to be embedded FRAM and DRAM, SRAM, and flash replacement, and to a lower extent standalone FRAM memory.^[89]^

**TSMC (Taiwan Semiconductor Manufacturing Company)**—As per their homepage and various conference proceedings, TSMC offer STT-MRAM and RRAM in production, with PCRAM and SOT-MRAM at more exploratory stages. Their 40 nm RRAM technology entered risk production at the end of 2017 and is available with a consumer-grade certified switching endurance of 10^4^ cycles, which is on a par with the low end of NAND flash. Mass production of the 40 nm and 28 nm RRAM nodes had commenced by 2022, whilst the 22 nm RRAM node was ready for production at that time.^[90]^ In 2024, TSMC presented a macro with RRAM integrated in a 12 nm node (smallest demonstrated CMOS integration amongst companies in this summary) with example parameters of 10^4^ write cycle endurance, 10 years retention at 105℃ (highest amongst companies in this summary), general operation from –40 to 125℃ for supply voltages of 0.6–0.9 V, word line voltages of >1.5 V, and read/write speeds of ca. 21 ns.^[91]^

TSMC STT-MRAM was available with reliability validation at 22 nm already at the end of 2018 and it completed reliability qualification on the 16 nm node in 2022 with 10^6^ endurance cycles,^[90]^ with further performance metrics presented in the IEDM 2023 contribution:^[92]^ operating voltages 0.7–1.98 V, operating temperatures −40 to 150℃, 20 years data retention at 150℃ (highest at this temperature amongst companies in this summary), standby currents of 67–107 µA, write currents of 22 mA, and a read speed of 6 ns for a read voltage of 0.68 V. In targeting SRAM replacement, STT-MRAM has been demonstrated in a 16 nm FinFET node in 2024 with read/write times of 7.5/20 ns for read voltages as low as 0.64 V (write voltage not provided), 10^12^ write endurance cycles, and 1 minute data retention at 125℃.^[93]^ Amongst other recent research progress are SOT-MRAM cells with switching speeds <2 ns at voltages of 1.5 V and extrapolated retention of 10 years.^[94]^

TSMC PCRAM development seems to be relatively a bit behind, with a 40 nm process reported in a research article in 2018.^[95]^ TSMC are also looking into ferroelectric HZO, and whilst an endurance performance of >10^11^ cycles was demonstrated in 2023,^[96]^ this work seems to be at a more exploratory stage than the other TSMC technologies. Further corporate research efforts are directed at finding two-terminal selector devices such as an arsenic-free GeCTe (germanium carbon telluride) selector with 10^11^ switching cycles together with a threshold voltage of ≈1.3 V and leakage current of ≈5nA.^[97]^

So far, the main target applications for TSMC RRAM and STT-MRAM are embedded memory and SRAM, but RRAM in-memory computing macros for AI have been demonstrated, too, such as an RRAM edge AI accelerator called CHIMERA.^[98]^ Other neural network investigations, some also based on PCRAM and MRAM, have been published, too, and Ref. 98 is just one recent example.

**UMC (United Microelectronics Corporation)**—As another globally leading semiconductor manufacturer from Taiwan besides TSMC, UMC qualified eMemory’s RRAM IP on UMC’s 22 nm ultra-low power process in 2023 as a 0.8/2.5 V platform with an endurance of 10^4^ cycles and 10 years data retention up to 105℃ and work on an even-lower-power 0.8/1.5 V platform is ongoing.^[99]^ 40 nm RRAM was qualified in 2021. Their main target application is embedded memory for IoT and automotive, but offers room for use for in-memory computing for AI applications, too.^[99]^ Whilst RRAM is the only technology featured on UMC’s homepage as a product, they are working on STT-MRAM with Avalanche Technology and the Avalanche STT-MRAM is qualified on UMC’s 22 nm platform as well since 2022.^[14]^ (Details see Avalanche.)

**Weebit Nano**—One of the leading startups in the area of eNVM, their chips are ready for production on SkyWater’s 130 nm CMOS with specifications of 1.8 V read voltage, 1.8 V + 3.3/3.6 V programming voltage, read access time <20 ns, 10 years retention, and 10^4^ endurance cycles, all at operating temperatures of −40 to 125℃. Qualification of the same 130 nm architecture for higher temperatures (150℃), endurance (10^5^), and other improved metrics are ongoing as of 2024, as are demonstrations of Weebit RRAM on GlobalFoundries’ 22 nm platform.^[24]^ Weebit has also demonstrated RRAM on CEA Leti’s 28 nm node in 2022 with >10^5^ cycles switching endurance and stable resistance for 15 h at 210℃.^[100]^ Weebit’s main targeted applications are embedded and standalone memory.

## Summary and outlook

Based on the current state of eNVM in industry, there is no eNVM ‘winner’ technology which seems poised to take a commanding lead in all areas of memory and AI applications. Whilst RRAM and MRAM are in a more advanced position, FRAM and PCRAM are still being pursued in industry, and if their outstanding challenges can be overcome, they may catch up to some extent, as fundamentally, they show great promise. For all four technologies, there exist some common generalisations about their strengths and weaknesses. Whilst some of them are indeed reflected in available products, they do not constitute fundamental roadblocks. For example, RRAM is generally said to have problems with cycling endurance and uniformity, but it is still one of the most widely used eNVM technologies for embedded memory in industry.

Extrapolating the current eNVM industry situation, we expect to see a continued coexistence of several eNVM technologies depending on application and cost. For example, MRAM is in the lead to replace SRAM and DRAM at some point due to its outstanding cycling endurance, whereas RRAM seems on a par with MRAM with respect to embedded flash replacement and has the potential advantage of cheaper manufacturing than MRAM. The latter must not be underestimated for actual end user products, as the example of the Micron/Intel 3D XPoint/Optane market entry and exit has demonstrated that for large-scale production, the economies of scale are more important in industry than technology performance. However, it is clear that eNVM now have a strong foothold in industry and we expect them to stay and grow in importance. We expect that first, embedded flash (NOR flash) will gradually be replaced, then SRAM and DRAM, and if the cost can be kept low (and this cannot be stressed enough), also standalone flash. We termed this ‘conventional memory replacement’ at the start of the manuscript, as opposed to the use of eNVM for in-memory computing and AI accelerator chips. This latter development is at a much earlier stage than conventional memory replacement, but when it will be realised at a large scale, we expect this to have a much stronger impact on the semiconductor, computing, and AI industry, even with revolutionary potential. This is because so far, AI is being implemented in Si CMOS, which is, however, not suitable because of the von Neumann/memory bottleneck. eNVM in-memory computing has the potential to overcome this and make AI hardware orders of magnitude faster and more efficient.

With the strong foothold of eNVM in conventional memory replacement in industry, for academic research, we recommend to be very clear about which questions are being addressed by new investigations and for them to be geared towards a clear scientific or technological end goal. Due to the advanced state of eNVM in industry, this will be hard for conventional storage applications, but there is a much wider field to explore with respect to in-memory, neuromorphic, bioelectronic, and AI hardware.

## Data Availability

As a large number of references for this article consist in web page accesses and datasheets, for future accountability, they are provided as snapshots in the University of Cambridge Apollo repository with DOI 10.17863/CAM.111155.
